# The Association of Different Types of Leisure Time Physical Activities with Cardiometabolic Outcomes in Singapore—Findings from the Multi-Ethnic Cohort Study

**DOI:** 10.3390/ijerph17239030

**Published:** 2020-12-03

**Authors:** Falk Müller-Riemenschneider, Yueheng Hong, Kristin Hui Xian Tan, Rob M. van Dam, Léonie Uijtdewilligen

**Affiliations:** 1 Saw Swee Hock School of Public Health, National University of Singapore, Singapore 117549, Singapore; ephhyh@nus.edu.sg (Y.H.); kristintanhx@gmail.com (K.H.X.T.); rob.van.dam@nus.edu.sg (R.M.v.D.); l.uijtdewilligen@vumc.nl (L.U.); 2 Yong Loo Lin School of Medicine, National University of Singapore, Singapore 117597, Singapore; 3 Digital Health Center, Berlin Institute of Health, 10117 Berlin, Germany; 4 Department of Nutrition, Harvard School of Public Health, Boston, MA 02115, USA

**Keywords:** exercise, observational study, body-mass index, hypertension, lipids

## Abstract

The study aimed to investigate the association between leisure time physical activity (LTPA) subtypes and cardiometabolic outcomes in the Singapore Multi-Ethnic Cohort (MEC). Self-reported data on socio-demographics, lifestyle factors, LTPA subtypes, and health screening data on body-mass index (BMI), waist circumference (WC), systolic and diastolic blood pressure (SBP and DBP), triglycerides (TG), and HDL-and LDL cholesterol were collected. Multivariable linear regression analyses were used adjusting for confounders. The mean age of 9768 participants was 45.2 ± 12.5 years (57.3% female, 47.3% Chinese, 26.0% Malay, and 26.8% Indians). Overall, 65.8% engaged in LTPA, and walking, strength/fitness and running were most common. Higher total LTPA was associated with lower WC, DBP, TG, a trend towards lower BMI, and higher SBP and HDL. Running was beneficially associated with all outcomes except for SBP and LDL. Balance exercises (BMI, SBP and DBP), cycling (BMI, WC and HDL), and strength/fitness (BMI, WC, TG and HDL) were also favorably associated with a number of outcomes, whereas ball games (DBP and TG), dancing (HDL) and other LTPA (DBP) were only favorably associated with selected outcomes. Unfavorable associations were found for total LTPA (SBP), strength/fitness (SBP), golf (DBP) and swimming (BMI and WC). Further research is warranted to inform future health promotion efforts.

## 1. Introduction

A lack of physical activity (PA) is a key risk factor for non-communicable diseases. The disease burden of physical inactivity is well-documented and comprises conclusive evidence of its association with, for example, type 2 diabetes, cardiovascular diseases, and different types of cancer [1,2,3,4]. However, at present, about 1/4 of the world population does not comply with PA recommendations and the World Health Organization (WHO) aims to reduce physical inactivity by 10% by 2025 [5,6].

While it is recognized that PA accumulated in various domains (e.g., household activities, or active transport) is beneficial for health, the largest body of evidence exists for leisure time PA (LTPA) [1,7,8]. However, there also appear to be some important differences related to specific aspects of LTPA. For instance, there is evidence for the increased health benefits of vigorous compared to moderate-intensity activity, probably due to greater physiological adaptations and greater associated increases in cardiorespiratory fitness [4,9,10]. In addition, the type of LTPA performed may have implications for subsequent health benefits. Thus, in addition to achieving recommended levels of overall PA, guidelines now also recommend engagement in specific types of LTPA, such as strength, flexibility and neuromotor exercises. The American College of Sports Medicine for instance, summarizes the specific health benefits of these activities, such as improved bone mass and bone strength, reduced risk of falls and improved flexibility [4,11]. Although epidemiological studies have most commonly investigated the health effects of overall PA, overall sports participation, or walking [12,13,14,15], there is also some evidence supporting differences in health-related outcomes depending on the type of LTPA. For instance, the Health Professional’s Follow-Up Study indicated that especially running, strength training and rowing were associated with a lower risk of coronary heart disease, but other LTPA types were not [16]. A cohort study from China has also reported beneficial associations between jogging, walking and tai chi and mortality, but not with engagement in other LTPA types [17]. However, another cross-sectional study from China compared a larger spectrum of LTPA types and found potential benefits in relation to the metabolic syndrome for jogging, tai chi and dancing, but not for other LTPA types [18].

A better understanding of the benefits of different LTPA types can be of relevance when tailoring PA promotion strategies to certain population groups. To strengthen the existing evidence, we conducted the current study based on data from the Singapore Multi-Ethnic Cohort (MEC) study. Our objectives were to investigate cross-sectional associations between total LTPA and ten LTPA types (i.e., balance exercises, ball games, cycling, dancing, golf, running, strength and fitness, swimming, walking, and other LTPA) and cardiometabolic health outcomes. We further investigated associations between LTPA subtypes and cardiometabolic outcomes according to the three major ethnic groups residing in Singapore: Chinese, Malays, and Indians.

## 2. Materials and Methods

### 2.1. Study Participants

The MEC study has been described elsewhere in detail [19]. Briefly, the MEC comprises a prospective cohort recruited between 2004 and 2010. Singapore citizens and permanent residents aged 21 years old and above could be included. Those with cancer, heart disease, stroke, renal failure and serious mental illness were excluded from participation. The MEC was formed by combining two existing cohorts: the Singapore Prospective Study Program (SP2) and the Singapore Cardiovascular Cohort Study (SCCS2). The SP2 and SCCS2 enrolled participants from four previous cross-sectional studies. These studies used random sampling of Singapore residents aged 18 years old and above, and disproportionate sampling stratified by ethnicity, to increase the numbers of ethnic minorities. A total of 6341 new participants were subsequently recruited through public outreach events at mosques, temples and community events, and referrals from existing cohort members. This finally resulted in a cohort of 14,815 participants. The study was approved by the National University of Singapore Institutional Review Board in Singapore (reference number 6–127).

### 2.2. Data Collection

All participants were visited by trained interviewers at their home. Information on socio-demographics, lifestyle (e.g., diet and PA), medication use, medical history, family history of diseases and health-related quality of life was collected as part of the interviewer-administered questionnaire. The participants were also invited to undergo a health screening [19,20].

### 2.3. Dependent and Independent Variables

PA was assessed with the SP2 PA questionnaire (SP2PAQ), which was adapted from several established questionnaires [20,21]. For the current study, only leisure time activities were considered. Participants indicated the number of times per week or per month and the duration in minutes that they engaged in any of the 48 different pre-defined leisure time activities. The 48 activities were then classified into ten LTPA subtypes: (1) balance exercises (e.g., Tai chi, Qi gong), (2) ball games (e.g., volleyball, tennis, football), (3) cycling, (4) dancing, (5) golf, (6) running, (7) strength and fitness (e.g., home exercises, weight lifting), (8) swimming, (9) walking (e.g., walking, hiking, mountain climbing) and (10) other LTPA (e.g., horseback riding, water skiing, bowling). Only activities of moderate intensity (3.0 metabolic equivalent of task (MET)) were taken into account [22].

Cardiometabolic outcomes included body mass index (BMI; in kg/m^2^), waist circumferences (in cm), systolic and DBP (in mmHg), high-density lipoprotein (HDL) and low-density lipoprotein (LDL) cholesterol (in mg/dL), and triglycerides (in mg/dL). All cardiometabolic measures were taken during the MEC health screening. Height and weight were assessed with a portable stadiometer and a SECA digital scale. Waist circumference was assessed with a stretch-resistant tape at the mid-point between the participant’s last rib and iliac crest. Participants rested for 5 min before their systolic and DBPs were measured twice with an automated digital monitor. A third reading was performed if the difference between the two readings of systolic blood pressure (SBP) or diastolic blood pressure (DBP) was greater than 10 or 5 mmHg, respectively. In case participant’s blood pressure exceeded the monitor range, a sphygmomanometer was used. Blood samples were taken from participants and were kept at the screening site at 4 °C. All samples were initially analyzed on the collection day at the biochemistry laboratory of the National University Hospital. Subsequent analyses were performed at the Singapore General Hospital. Both laboratories are accredited by the College of American Pathologists. For the purpose of the current analysis, Triglycerides, HDL and LDL cholesterol were investigated [19].

Participants were excluded from the present analyses if they did not provide information in the PA section of the MEC interviewer-administered questionnaire or did not undergo the health screening. Additionally, we also excluded participants who had a medical history of cardiovascular diseases, cancer, and diabetes mellitus, or were pregnant at times of the data collection for the MEC. Participants who were not of Chinese, Malay or Indian ethnicity were also excluded.

### 2.4. Statistical Analysis

Descriptive statistics (mean and standard deviation (SD) and proportions), were derived for socio-demographic variables. ANOVA and chi-square tests (for continuous and categorical variables) were used to compare Chinese, Malays and Indians.

We dichotomized each LTPA subtype based on whether a participant had engaged in this activity or not (yes/no). Time spent in LTPA subtypes in hours/week, and in MET-h/week was calculated. Total LTPA was calculated by summing the values of all ten LTPA subtypes. Total LTPA showed an approximate mean of 15 MET-h/week, and was therefore categorized into 0 MET-h/week, >0–15 MET-h/week and >15 MET-h/week.

Linear regression analyses were used to examine associations of LTPA subtypes and total LTPA with continuous cardiometabolic outcomes. Analyses were adjusted for age, gender, ethnicity, education status, smoking status, alcohol consumption and the other nine LTPA subtypes. Analyses pertaining to blood pressure and cholesterol outcomes were additionally adjusted for BMI. Stratified analyses for ethnicity were performed, interactions between total LTPA categories and ethnicity were analyzed. All statistical analyses were done using R Version 3.3.1 [23]. For all analyses, two-sided statistical significance was set at *p* < 0.05.

## 3. Results

A total of 9768 participants (mean age 45.2 ± 12.5 years; 57.3% females) were included in the analytic sample. Ethnic distribution was as follows: 4618 (47.3%) participants were Chinese, 2535 (26.0%) participants were Malays, and 2615 (26.8%) participants were Indians. Other socio-demographic characteristics for the sample and according to ethnicity are presented in Table 1. Statistically significant differences between ethnic groups were observed for all socio-demographic characteristics (*p* < 0.001 for all variables, except for age with *p* = 0.003).

**Figure 1:**
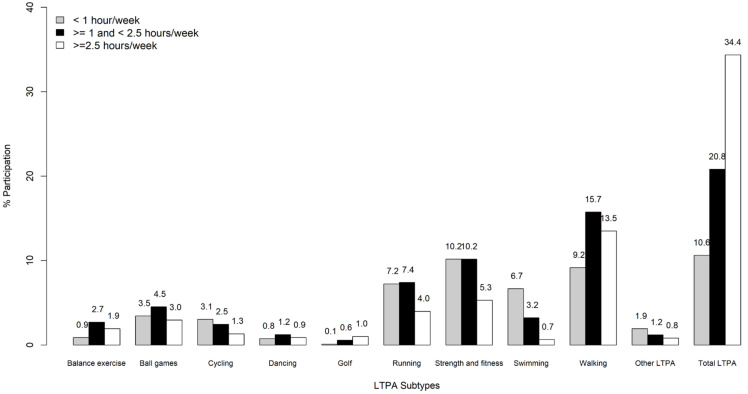
Proportion of duration of participation among various leisure time physical activities.

**Figure 2:**
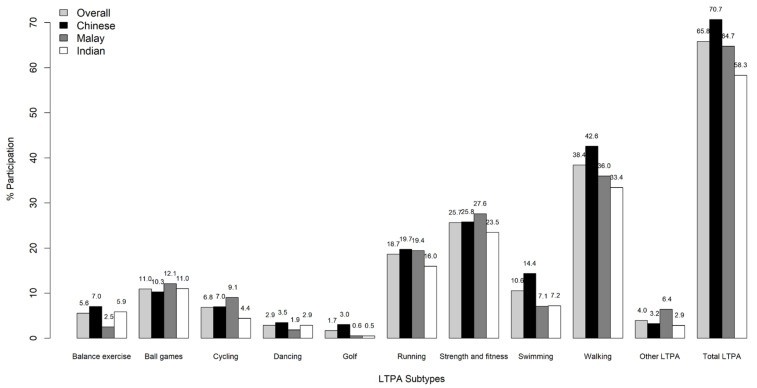
Proportion of participation in various leisure time physical activities across different ethnic groups.

Overall, 65.8% of the sample reported to engage in LTPA (Figure 1 and Figure 2). The proportion of participants engaging in any LTPA was highest among Chinese (70.7%), followed by Malays (64.7%) and Indians (58.3%). Among these participants, 10.6% reported engaging less than 1 h on a weekly basis, while 20.8% spent between 1 and 2.5 h/week, and 34.4% spent more than 2.5 h/week in total LTPA. Walking, strength and fitness, and running were the three most commonly reported subtypes of LTPA, and this was consistent across all three ethnic groups (Figure 2). However, differences across ethnic groups were also noted. For instance, the highest prevalence of walking (42.6%), swimming (14.4%), balance exercises (7.0%), dancing (3.6%), and golf (3.0%) was reported by Chinese, while Malays reported the highest prevalence for strength and fitness (27.6%), ball games (12.1%), cycling (9.1%), and other LTPA (6.4%). The prevalence of running was almost identical for Chinese (19.7%) and Malays (19.4%). Indians did not report the highest prevalence for any LTPA subtype.

### 3.1. Association of Total LTPA with Cardiometabolic Outcomes

There was a trend towards lower BMI with higher total LTPA (Table 2). Moreover, total LTPA was associated with a lower waist circumference, lower DBP, lower triglyceride levels, higher SBP and HDL-cholesterol. When adjusted for BMI, effect sizes for SBP became more pronounced, while those for DBP, triglyceridesand HDL-cholesterol were somewhat attenuated.

### 3.2. Association of LTPA Subtypes with Cardiometabolic Outcomes

Table 3 presents the associations of participation in the ten LTPA subtypes with investigated outcomes.

Participation in balance exercise, cycling, running, and strength and fitness were associated with lower BMI, while swimming was associated with higher BMI. Swimming was also associated with a higher waist circumference. Cycling, running, and strength and fitness, on the other hand, were found to be associated with lower waist circumference.

Those engaging in balance exercises had lower SBP and DBP. Ball games, running and other LTPA were associated with lower DBP. Engaging in strength and fitness exercises was associated with higher SBP. Similarly, golf was associated with a higher DBP, and walking was borderline significantly associated with higher DBP.

Ball games, running, and strength and fitness were associated with lower triglycerides. Cycling, dancing, running, and strength and fitness were associated with higher HDL-cholesterol. No significant associations between any LTPA subtype and LDL-cholesterol were found.

In sum, participation in running was most consistently and beneficially associated with outcomes except for SBP and LDL cholesterol, even after additional adjustment for BMI. Balance exercises (BMI, systolic and DBP), cycling (BMI, waist circumference and HDL cholesterol), as well as strength and fitness (BMI, waist circumference, triglycerides and HDL cholesterol) were also favorably associated with a number of outcomes, whereas ball games (DBP and triglycerides), dancing (HDL cholesterol) and other LTPA (DBP) were only favorably associated with selected outcomes. In addition to its beneficial associations, strength and fitness was associated with higher SBP. Golf (DBP) and swimming (BMI and waist circumference) were unfavorably associated with selected outcomes and, apart from that, showed no favorable associations. Walking was not associated with any of the investigated outcomes. The results remained largely stable when adjusting for BMI, but effect sizes were attenuated somewhat.

Investigating associations between total LTPA and cardiometabolic outcomes, statistically significant interactions with ethnicity were observed with regard to BMI, WC, and TG. In the stratified analyses (Supplementary Table S1), total LTPA was beneficially associated with HDL among Chinese and Indians, but not Malay. Only among Indians was total LTPA associated with BMI, WC, SDP (detrimentally), and TG. When investigating LTPA subtypes (Supplementary Table S2), less statistically significant associations than in the overall sample were observed. Among Chinese, cycling was associated with lower BMI, triglycerides and higher HDL cholesterol. Running was associated with lower DBP and lower triglycerides. Balance exercises were associated with lower BMI and DBP. Dancing was associated with higher HDL cholesterol and strength and fitness was associated with both higher HDL cholesterol and SBP. Swimming was associated with higher BMI and DBP, whereas walking was associated with higher triglycerides. Among Malays, running was associated with all outcomes, apart from waist circumference and SBP. Otherwise, only balance exercise and ball games were associated with lower DBP. Swimming, on the other hand, was associated with an increased waist circumference. Among Indians, running was associated with lower BMI, and strength and fitness exercises with lower triglyceride levels and higher HDL-cholesterol. Balance exercises were associated with lower HDL-cholesterol, and golf with higher DBP.

## 4. Discussion

In this large cross-sectional study of a multi-ethnic Asian population residing in Singapore, we found that about two-thirds of the population engage in LTPA weekly. Engagement in LTPA was considerably higher among Chinese as compared to Malays and especially Indians. Despite some differences in preferred LTPA subtypes, walking, strength and fitness, and running were the most frequently performed LTPA subtypes across all ethnic groups. The findings of this study also revealed beneficial associations between total LTPA and waist circumference, DBP, triglycerides, and HDL cholesterol, and to a certain extent BMI, but not with SBP and LDL cholesterol. Important differences between LTPA subtypes and their association with cardiometabolic outcomes emerged. In particular, running, and to a somewhat lesser extent, balance exercises and cycling, were favorably associated with cardiometabolic outcomes. Strength and fitness exercises were also favorably associated with a number of outcomes, but were also associated with higher SBP. For other LTPA subtypes, including ball games and dancing, there was very limited evidence for favorable associations. In the case of walking, golf and swimming, none or even adverse associations with investigated outcomes were observed. We also noted some evidence for differences in investigated associations across ethnic groups. For instance, associations between total LTPA and BMI, WC, and TG were only observed among Indians. Beneficial effects of running appeared to be particularly consistent among Malays. Similar observations were made for cycling among Chinese. In terms of LTPA subtypes, few significant beneficial associations were noted for Indians, and they related mostly to running and strength and fitness exercises.

Our findings are generally consistent with the large body of evidence supporting beneficial associations between LTPA and various clinical health outcomes, as well as mortality [2,7,8,24]. Fewer studies have investigated different LTPA types and their association with health outcomes, [14,16,17,18] and we noted some similarities but also differences compared to the existing evidence. For instance, previous studies have also reported beneficial effects of running, strength training, and balance exercises such as Tai Chi [16,17,18,25]. These findings are similar to our observations, but by directly comparing a range of LTPA subtypes in one study, our findings suggest a greater consistency in these beneficial associations for running. While dancing was previously reported to be favorably associated with metabolic syndrome in a study from China, this was not consistently the case in our study [18]. Walking is probably the most frequently investigated LTPA subtype [13,15,17,18]. While reports about the health benefits of walking in past studies were not always consistent, recent systematic reviews and meta-analysis of observational and intervention studies have provided further evidence about the health benefits of engaging in walking [13,26]. These results conflict with the lack of beneficial associations observed in our study, which may be related to different factors. Importantly, past studies have reported that walking intensity is more strongly associated with health outcomes than walking volume [13,27]. However, a recent population-based study from Singapore, investigating walking intensity in terms of stepping cadence, highlighted that the large majority of walking (stepping) in Singapore is of very low intensity [28]. Another possible reason for differences may be related to the volume of walking. In our study, the median time spend walking was only 1.7 h per week. Many previous studies that reported health benefits of walking, however, had considered greater volumes of walking activity [13,14,15]. At present, the evidence for the health benefits of walking is largely based on studies from other regions of the world. Considering that walking is the cornerstone of PA promotion around the world, this warrants further investigation of the health benefits of walking in South-East Asia.

Observed differences in associations between LTPA subtypes and health outcomes may, in part, be related to their dominant physiological response, for instance, in terms of muscle strengthening, neuromotor skills, or cardio-respiratory fitness [4,29]. Since our study only focused on cardiometabolic outcomes, LTPA subtypes most closely related to the cardiorespiratory system, such as running, may show stronger associations. On the other hand, the specific beneficial aspects of some exercises that are unrelated to cardiometabolic outcomes may not have been captured. For example, the benefits of neuromotor exercises on fall risks or the benefits of strength training on bone mass and fracture risk [4]. LTPA subtypes, such as ball games or dancing may also offer specific benefits due to their social interaction, which are also unlikely to be observed when only investigating cardiometabolic outcomes. Variation across LTPA subtypes could also be the result of differences in activity-related energy expenditure, which is a function of exercise duration and intensity. In our study, the duration of engagement tended to be higher for LTPA subtypes that are commonly considered to be of lower intensity (e.g., golf, balance exercise, or walking) as compared to higher intensity LTPA, such as running [22]. This may mitigate the higher energy expenditure of higher intensity LTPA subtypes. Moreover, studies have shown that vigorous activities (e.g., running) convey greater health benefits than less intensive activities, even after controlling for differences in energy expenditure, possibly due to greater physiological adaptations [9,30]. Differences across LTPA types have also been reported after taking total energy expenditure into consideration [18]. Another explanation for differences between LTPA subtypes may be related to the continuity of certain exercises. While activities such as running or Tai Chi tend to be continuous, other exercise types, such as playing golf or other ball games, are more frequently interrupted. Thus, although individuals report to spend more time on some activities, such as golf as compared to running, this may not be the case for the ‘net’ exercise time. 

Previous reviews have emphasized the scarcity of studies investigating the health benefits of physical activity in south Asian populations, and especially the lack of head-to-head comparisons across different ethnic groups [31]. A unique feature of our study is the fact that it comprises a large population-based sample conducted in a multi-ethnic Asian population of Chinese, Malay and Indian background. Considerable differences in the volume and type of LTPA between ethnic groups were observed, which is broadly consistent with prior research from Singapore and other countries [28,31,32]. While few studies have directly compared different Asian ethnic groups, some evidence exists to support our observations that associations between LTPA and LTPA subtypes with cardiometabolic outcomes may differ according to ethnic group [31,33]. However, the reasons for these differences are complex and warrant further investigation in future longitudinal studies.

Despite its strength, the limitations of our study need to be acknowledged. They include the cross-sectional study design. Although we attempted to minimize the risk of reverse causality, this concern cannot be ruled out entirely. Secondly, our assessment of PA relies on self-report. While LTPA types cannot currently be easily differentiated by objective measurements, self-report is prone to bias. Thirdly, in investigating LTPA subtypes our analysis is limited by the small percentage and limited time participants engaged in these activities. For balance exercises, dancing, golf, swimming, and other LTPA, less than 5% of the study population reported to engage in more than 1 h per week. For swimming, the median time per week was only 0.5 h. It is also possible that exercises are not performed at their usual intensity levels. For instance, swimming pools are widely available in Singapore due to the hot and humid climate, but their use and reported swimming time may not always be at the usual intensity level. Finally, excluding participants with medical history and those who were not of Chinese, Malay, or Indian ethnicity could reduce the generalizability of our findings.

## 5. Conclusions

Our study in a multi-ethnic Asian population confirms beneficial associations between LTPA and cardiometabolic outcomes, but also indicates some possible differences across ethnic groups. Beneficial effects may differ across LTPA subtypes and in relation to investigated outcomes. They were most pronounced for running, strength and fitness and balance exercises, which warrants further investigation into the diverse health benefits of different types of LTPA in order to guide health promotion in South-East Asia.

## Figures and Tables

**Table 1:** Profile of participants (N = 9768).

Characteristics	All (N = 9768)		Chinese (N = 4618)		Malays (N = 2535)		Indians (N = 2615)		
	N	Proportion (%)	N	Proportion (%)	N	Proportion (%)	N	Proportion (%)	*p*-Value for between Ethnicity Diffterences
Age (in years), mean ± SD	45.2 ± 12.5		46.3 ± 12.4		44.3 ± 12.7		44.3 ± 12.5		**<0.001**
Gender									
Male	4169	42.7	2054	44.5	1037	40.9	1078	41.2	**0.003**
Female	5599	57.3	2564	55.5	1498	59.1	1537	58.8	
Marital Status (Missing = 4)									
Never married	1624	16.6	882	19.1	352	13.9	390	14.9	**<0.001**
Currently married	7428	76.1	3493	75.6	1981	78.1	1954	74.8	
Separated	43	0.4	13	0.3	14	0.6	16	0.6	
Divorced	293	3.0	91	2.0	86	3.4	116	4.4	
Widowed	376	3.9	137	3.0	101	4.0	138	5.3	
Educational Status									
No formal education/Lower primary	687	7.0	302	6.5	191	7.5	194	7.4	**<0.001**
Primary education	1966	20.1	733	15.9	609	24.0	624	23.9	
Secondary education	3558	36.4	1549	33.5	1076	42.4	933	35.7	
Technical certification ^a^	587	6.0	162	3.5	273	10.8	152	5.8	
Foundation/Associate degrees ^b^	1707	17.5	999	21.6	314	12.4	394	15.1	
University	1263	12.9	873	18.9	72	2.8	318	12.2	
Employment Status (Missing = 3)									
Working	6451	66.1	3294	71.4	1514	59.7	1643	62.8	**<0.001**
Student (full-time)	215	2.2	118	2.6	37	1.5	60	2.3	
Homemaker/Housewife	2171	22.2	762	16.5	776	30.6	633	24.2	
Retired	526	5.4	314	6.8	115	4.5	97	3.7	
Unemployed (able to work)	312	3.2	89	1.9	79	3.1	144	5.5	
Unemployed (unable to work)	50	0.5	16	0.3	6	0.2	28	1.1	
Others	40	0.4	22	0.5	8	0.3	10	0.4	
Mean Monthly Household Income (SGD)									
Less than 2000	2205	22.6	633	13.7	775	30.6	797	30.5	**<0.001**
2000 to 3999	2471	25.3	916	19.8	745	29.4	810	31.0	
4000 to 5999	1561	16.0	684	14.8	428	16.9	449	17.2	
6000 to 9999	989	10.1	570	12.3	176	6.9	243	9.3	
More than 10,000	377	3.9	314	6.8	14	0.6	49	1.9	
Missing	2165	22.2	1501	32.5	397	15.7	267	10.2	

^a^ Includes technical schools for pre-employment—in Singapore terms: ITE, Institute of Technical Education /NTC, National technical certificate. ^b^ Includes—in Singapore terms: ‘A’ level/Polytechnic/Diploma. Bold values indicate statistical significance.

**Table 2:** Association between cardiometabolic measures and total leisure time physical activity (LTPA).

Total LTPA	LTPA	Outcomes						
		BMI (kg/m^2^)	Waist Circumference (cm)	Systolic Blood Pressure (mmHg)	Diastolic Blood Pressure (mmHg)	Triglycerides (mg/dL)	HDL (mg/dL)	LDL (mg/dL)
		Effect Size (95% CI)	Effect Size (95% CI)	Effect Size (95% CI)	Effect Size (95% CI)	Effect Size (95% CI)	Effect Size (95% CI)	Effect Size (95% CI)
Model 1 *	0 MET-h/wk	reference	reference	reference	reference	reference	reference	reference
	0–15 MET-h/wk	−0.28 (−0.50, 0.07) *p* = 0.010	−1.55 (−2.72, −0.37) *p* = 0.010	0.56 (−0.26, 1.39) *p* = 0.181	−0.001 (−0.49, 0.49) *p* = 0.997	−3.39 (−7.03, 0.25) *p* = 0.068	0.78 (0.18, 1.37) *p* = 0.011	0.40 (−1.15, 1.96) *p* = 0.611
	> 15 MET-h/wk	−0.23 (−0.48, 0.02) *p* = 0.067	−1.44 (−2.77, −0.10) *p* = 0.035	1.35 (0.41, 2.29) *p* = 0.005	−0.54 (−1.10, 0.01) *p* = 0.055	−6.19 (−10.34, −2.03) *p* = 0.004	1.83 (1.15, 2.51) P < 0.001	0.17 (−1.61, 1.95) *p* = 0.853
	Test for Trend	*p* = 0.146	*p* = 0.058	*p* = 0.011	*p* = 0.005	*p* < 0.001	*p* < 0.001	*p* = 0.317
Model 2 ^	0 MET-h/wk			reference	reference	reference	reference	reference
	0–15 MET-h/wk			0.84 (0.05, 1.64) *p* = 0.038	0.14 (−0.34, 0.61) *p* = 0.575	−2.15 (−5.68, 1.38) *p* = 0.232	0.52 (−0.05, 1.08) *p* = 0.072	0.76 (−0.79. 2.30) *p* = 0.338
	> 15 MET-h/wk			1.54 (0.63, 2.45) *p* = 0.001	−0.45 (−0.99, 0.09) *p* = 0.104	−5.24 (−9.26, −1.21) *p* = 0.011	1.63 (0.98, 2.27) *p* < 0.001	0.44 (−1.32, 2.21) *p* = 0.622
	Test for Trend			*p* = 0.003	*p* = 0.011	*p* = 0.002	*p* < 0.001	*p* = 0.437

* Model adjusted for age, gender, ethnicity, educational status, smoking status, alcohol consumption and total LTPA. ^: Additional BMI adjustment for systolic and diastolic blood pressure, triglycerides, HDL and LDL. CI, confidence interval; cm, centimeters; BMI, body mass index; HDL, high-density lipoprotein; kg/m^2^, kilogram–meter square; LDL, low-density lipoprotein; LTPA, leisure time physical activity; MET, metabolic equivalent of task; mg/dL, milligrams per deciliter; mmHG, millimeters of mercury; h/wk, hours per week. Bold values indicate statistical significance.

**Table 3:** Association between cardiometabolic measures and leisure time physical activity (LTPA) subtypes.

LTPA Subtypes		Outcomes						
		BMI (kg/m^2^)	Waist Circumference (cm)	Systolic Blood Pressure (mmHg)	Diastolic Blood Pressure (mmHg)	Triglycerides (mg/dL)	HDL (mg/dL)	LDL (mg/dL)
Categorized as Participated Yes/No with No Being the Reference Category		Effect Size (95% CI)	Effect Size (95% CI)	Effect Size (95% CI)	Effect Size (95% CI)	Effect Size (95% CI)	Effect Size (95% CI)	Effect Size (95% CI)
Balance exercises	Model 1 *	−0.75 (−1.15, −0.35) *p* < 0.001	−1.24 (−3.43, 0.95) *p* = 0.269	−2.06 (−3.63, −0.49) *p* = 0.010	−1.60 (−2.53, −0.67) *p* = 0.001	−1.56 (−8.46, 5.34) *p* = 0.657	−0.75 (−1.88, 0.39) *p* = 0.196	−1.91 (−4.88, 1.05) *p* = 0.206
	Model 2 ^			−1.38 (−2.90, 0.14) *p* = 0.076	−1.25 (−2.16, −0.34) *p* = 0.007	1.36 (−5.33, 8.06) *p* = 0.690	−1.38 (−2.46, −0.31) *p* = 0.012	−1.05 (−3.98, 1.89) *p* = 0.485
Ball games	Model 1 *	0.13 (−0.19, 0.44) *p* = 0.435	−1.42 (−3.14, 0.29) *p* = 0.104	0.02 (−1.15, 1.20) *p* = 0.971	−0.97 (−1.66, −0.27) *p* = 0.006	−5.49 (−10.73, −0.26) *p* = 0.040	0.18 (−0.68, 1.04) *p* = 0.686	1.15 (−1.10, 3.40) *p* = 0.317
	Model 2 ^			−0.10 (−1.23, 1.04) *p* = 0.869	−1.02 (−1.70, −0.34) *p* = 0.003	−5.94 (−11.02, −0.86) *p* = 0.022	0.27 (−0.54, 1.09) *p* = 0.512	1.00 (−1.23, 3.22) *p* = 0.379
Cycling	Model 1 *	−0.59 (−0.97, −0.22) *p* = 0.002	−2.08 (−4.11, −0.06) *p* = 0.044	−1.00 (−2.39, 0.39) *p* =0.159	−0.24 (−1.06, 0.58) *p* = 0.566	−3.47 (−9.66, 2.72) *p* = 0.271	1.16 (0.15, 2.18) *p* = 0.025	−0.85 (−3.50, 1.80) *p* = 0.530
	Model 2 ^			−0.56 (−1.90, 0.79) *p* = 0.416	−0.03 (−0.83, 0.78) *p* = 0.943	−1.09 (−7.09, 4.91) *p* = 0.723	0.64 (−0.32, 1.61) *p* = 0.189	−0.27 (−2.89, 2.36) *p* =0.842
Dancing	Model 1 *	0.10 (−0.44, 0.65) *p* = 0.711	−0.53 (−3.50, 2.45) *p* = 0.729	−0.15 (−2.25, 1.96) *p* = 0.891	−0.43 (−1.67, 0.81) *p* = 0.497	1.10 (−8.15, 10.34) *p* = 0.816	1.67 (0.16, 3.19) *p* = 0.031	−0.45 (−4.42, 3.52) *p* = 0.824
	Model 2 ^			−0.23 (−2.26, 1.81) *p* = 0.827	−0.47 (−1.69, 0.74) *p* = 0.448	0.11 (−8.86, 9.07) *p* = 0.982	1.88 (0.44, 3.31) *p* = 0.010	−0.72 (−4.65, 3.21) *p* = 0.719
Golf	Model 1 *	0.49 (−0.22, 1.20) *p* = 0.177	1.78 (−2.08, 5.65) *p* = 0.367	−0.38 (−3.09, 2.34) *p* = 0.786	2.04 (0.44, 3.64) *p* = 0.013	6.55 (−5.90, 18.99) *p* = 0.303	0.14 (−1.90, 2.19) *p* = 0.892	3.25 (−2.10, 8.60) *p* = 0.234
	Model 2 ^			−0.77 (−3.39, 1.86) *p* = 0.567	1.85 (0.28, 3.42) *p* = 0.021	4.54 (−7.52, 16.61) *p* = 0.461	0.56 (−1.37, 2.50) *p* = 0.568	2.76 (−2.53, 8.06) *p* = 0.307
Running	Model 1 *	−0.41 (−0.66, −0.15) *p* = 0.002	−1.47 (−2.86, −0.08) *p* = 0.038	−0.19 (−1.15, 0.77) *p* = 0.702	−1.01 (−1.58, −0.45) *p* < 0.001	−9.44 (−13.70, −5.18) *p* < 0.001	0.81 (0.11, 1.51) *p* = 0.023	−1.69 (−3.52, 0.14) *p* = 0.071
	Model 2 ^			0.12 (−0.81, 1.05) *p* = 0.803	−0.87 (−1.42, −0.31) *p* = 0.002	−7.71 (−11.84, −3.57) *p* < 0.001	0.44 (−0.22, 1.10) *p* = 0.192	−1.26 (−3.07, 0.55) *p* = 0.174
Strength and fitness	Model 1 *	−0.25 (−0.47, −0.03) *p* = 0.027	−1.20 (−2.39, 0) *p* = 0.050	1.39 (0.55, 2.23) *p* = 0.001	−0.08 (−0.58, 0.42) *p* = 0.748	−4.13 (−7.84, −0.42) *p* = 0.029	1.68 (1.08, 2.29) *p* < 0.001	0.48 (−1.12, 2.07) *p* = 0.558
	Model 2 ^			1.62 (0.81, 2.44) *p* < 0.001	0.03 (−0.46, 0.52) *p* = 0.908	−3.18 (−6.78, 0.42) *p* = 0.083	1.46 (0.89, 2.04) *p* < 0.001	0.75 (−0.83, 2.33) *p* = 0.351
Swimming	Model 1 *	0.64 (0.33, 0.96) *p* < 0.001	2.06 (0.36, 3.76) *p* = 0.018	0.15 (−1.02, 1.33) *p* = 0.800	0.54 (−0.15, 1.23) *p* = 0.128	4.66 (−0.57, 9.89) *p* = 0.080	−0.51 (−1.37, 0.35) *p* = 0.244	1.42 (−0.82, 3.67) *p* = 0.213
	Model 2 ^			−0.52 (−1.66, 0.62) *p* = 0.370	0.22 (−0.46, 0.90) *p* = 0.526	1.88 (−3.20, 6.95) *p* = 0.469	0.08 (−0.73, 0.90) *p* = 0.843	0.74 (−1.49, 2.96) *p* = 0.516
Walking	Model 1 *	−0.04 (−0.23, 0.16) *p* = 0.709	−0.21 (−1.26, 0.84) *p* = 0.695	0.22 (−0.51, 0.96) *p* = 0.551	0.39 (−0.05, 0.82) *p* = 0.082	1.10 (−2.16, 4.36) *p* = 0.508	0.41 (−0.13, 0.94) *p* = 0.137	−0.26 (−1.66, 1.14) *p* = 0.718
	Model 2 ^			0.30 (−0.42, 1.01) *p* = 0.416	0.42 (−0.01, 0.84) *p* = 0.056	1.26 (−1.90, 4.42) *p* = 0.435	0.37 (−0.13, 0.88) *p* = 0.150	−0.20 (−1.59, 1.18) *p* = 0.773
Other LTPA	Model 1 *	0.19 (−0.30, 0.67) *p* = 0.449	1.03 (−1.59, 3.66) *p* = 0.441	−0.32 (−2.13, 1.49) *p* = 0.731	−1.08 (−2.15, −0.02) *p* = 0.047	−3.55 (−11.59, 4.48) *p* = 0.386	0.51 (−0.81, 1.83) *p* = 0.452	0.27 (−3.18, 3.71) *p* = 0.880
	Model 2 ^			−0.50 (−2.24, 1.25) *p* = 0.578	−1.17 (−2.21, −0.12) *p* = 0.029	−4.30 (−12.10, 3.50) *p* = 0.280	0.72 (−0.53, 1.97) *p* = 0.257	0.003 (−3.41, 3.42) *p* = 0.999

* Model adjusted for age, gender, ethnicity, educational status, smoking status, alcohol consumption and other respective LTPA subtypes. ^: Additional BMI adjustment for systolic and diastolic blood pressure, triglycerides, HDL and LDL. CI, confidence interval; BMI, body mass index; HDL, high-density lipoprotein; LDL, low-density lipoprotein; LTPA, leisure time physical activity. Bold values indicate statistical significance.

## Supplementary Materials

The following are available online at https://www.mdpi.com/1660-4601/17/23/9030/s1. Table S1: Association between cardiometabolic measures and total leisure time physical activity (LTPA), stratified by ethnicity; Table S2: Association between cadiometabolic measures and leisure time physical activity (LTPA) subtypes, stratified by ethnicity.
