# Supplementary material for: The Association of Different Types of Leisure Time Physical Activities with Cardiometabolic Outcomes in Singapore—Findings from the Multi-Ethnic Cohort Study
Source: Int J Environ Res Public Health. 2020 Dec 3;17(23):9030. doi: 10.3390/ijerph17239030 (PMC7731378; doi:10.3390/ijerph17239030)
Supplement: Supplementary file 1 [file ijerph-17-09030-s001.pdf]

Supplemental table 1: Association between cardiometabolic measures and total leisure time physical activity (LTPA), stratified by ethnicity.

			Outcomes						
	Ethnicity	Total LTPA	BMI (kg/m²)	Waist Circumference (cm)	Systolic Blood Pressure (mmHg)	Diastolic Blood Pressure (mmHg)	Triglycerides (mg/dL)	HDL (mg/dL)	LDL (mg/dL)
			**Effect size (95%CI)**	**Effect size (95%CI)**	**Effect size (95%CI)**	**Effect size (95%CI)**	**Effect size (95%CI)**	**Effect size (95%CI)**	**Effect size (95%CI)**
Model 1*	Chinese	0 MET-h/wk	reference	reference	reference	reference	reference	reference	reference
		0-15 MET-h/wk	-0.24 (-0.50, 0.02) P=0.065	-0.48 (-1.58, 0.62) P=0.395	0.75 (-0.46, 1.95) P=0.224	-0.04 (-0.68, 0.77) P=0.904	1.51 (-3.24, 6.25) P=0.534	0.30 (-0.64, 1.23) P=0.536	0.30 (-1.92, 2.52) P=0.790
		> 15 MET-h/wk	-0.04 (-0.34, 0.25) P=0.769	-0.03 (-1.3, 1.23) P=0.962	0.92 (-0.46, 2.31) P=0.192	-0.65 (-1.49, 0.19) P=0.130	-1.75 (-7.24, 3.74) P=0.531	**1.77 (0.69, 2.86) P=0.001**	1.67 (-0.90, 4.24) P=0.202
	Malay	0 MET-h/wk	reference	reference	reference	reference	reference	reference	reference
		0-15 MET-h/wk	-0.10 (-0.57, 0.38) P=0.687	-1.29 (-4.27, 1.7) P=0.398	0.38 (-1.22, 1.98) P=0.644	-0.08 (-1.03, 0.87) P=0.866	-1.34 (-8.81, 6.13) P=0.726	0.97 (-0.13, 2.07) P=0.084	0.85 (-2.36, 4.06) P=0.602
		> 15 MET-h/wk	-0.08 (-0.62, 0.46) P=0.774	-0.90 (-4.27, 2.48) P=0.603	1.74 (-0.07, 3.55) P=0.060	-0.36 (-1.43, 0.71) P=0.511	-5.13 (-13.58, 3.33) P=0.235	1.03 (-0.21, 2.27) P=0.103	-0.79 (-4.42, 2.84) P=0.669
	Indian	0 MET-h/wk	reference	reference	reference	reference	reference	reference	reference
		0-15 MET-h/wk	**-0.53 (-0.99, -0.06) P=0.026**	**-3.18 (-5.81, -0.55) P=0.018**	0.33 (-1.27, 1.93) P=0.683	0.11 (-0.8, 1.02) P=0.811	**-13.2 (-21.05, -5.34) P=0.001**	**1.48 (0.41, 2.55) P=0.007**	0.65 (-2.35, 3.65) P=0.669
		> 15 MET-h/wk	**-0.56 (-1.08, -0.03) P=0.038**	**-3.71 (-6.68, -0.74) P=0.014**	**2.06 (0.25, 3.87) P=0.026**	-0.13 (-1.16, 0.90) P=0.807	**-12.63 (-21.52, -3.73) P=0.005**	**2.46 (1.25, 3.67) P=<0.001**	-1.56 (-4.96, 1.84) P=0.369
Model	Chinese	0 MET-h/wk			reference	reference	reference	reference	reference

2^		0-15 MET-h/wk		1.04 (-0.12, 2.2) P=0.078	0.18 (-0.53, 0.90) P=0.611	2.93 (-1.60, 7.46) P=0.206	-0.05 (-0.92, 0.83) P=0.916	0.69 (-1.50, 2.88) P=0.536
		> 15 MET-h/wk		0.92 (-0.41, 2.26) P=0.176	-0.66 (-1.48, 0.16) P=0.117	-1.40 (-6.64, 3.84) P=0.600	1.70 (0.68, 2.71) P=0.001	1.74 (-0.79, 4.27) P=0.178
	Malay	0 MET-h/wk		reference	reference	reference	reference	reference
		0-15 MET-h/wk		0.48 (-1.07, 2.02) P=0.544	-0.03 (-0.95, 0.89) P=0.948	-0.43 (-7.60, 6.74) P=0.907	0.84 (-0.19, 1.87) P=0.111	1.12 (-2.06, 4.29) P=0.490
		> 15 MET-h/wk		**1.80 (0.05, 3.55) P=0.044**	-0.33 (-1.38, 0.72) P=0.538	-4.65 (-12.77, 3.46) P=0.261	0.98 (-0.18, 2.15) P=0.099	-0.63 (-4.22, 2.96) P=0.732
	Indian	0 MET-h/wk		reference	reference	reference	reference	reference
		0-15 MET-h/wk		0.75 (-0.81, 2.31) P=0.343	0.31 (-0.59, 1.20) P=0.505	**-11.81 (-19.57, -4.06) P=0.003**	**1.19 (0.16, 2.22) P=0.023**	1.07 (-1.91, 4.06) P=0.481
		> 15 MET-h/wk		**2.48 (0.72, 4.24) P=0.006**	0.07 (-0.94, 1.09) P=0.889	**-11.15 (-19.93, -2.37) P=0.013**	**2.08 (0.92, 3.25) P=<0.001**	-1.07 (-4.45, 2.31) P=0.537

*: Model adjusted for age, gender, ethnicity, educational status, smoking status, alcohol consumption and total LTPA. ^: Additional BMI adjustment for systolic and diastolic blood pressure, triglycerides, HDL and LDL. CI, confidence interval; cm, centimeters; BMI, body mass index; HDL, high-density lipoprotein; kg/m^2, kilogram-meter square; LDL, low-density lipoprotein; LTPA, leisure time physical activity; MET, metabolic equivalent of task; mg/dL, milligrams per deciliter; mmHG, millimeters of mercury; h/wk, hours per week. Bold values indicate statistical significance.

Supplemental table 2: Association* between cardiometabolic measures and leisure time physical activity (LTPA) subtypes, stratified by ethnicity.

[redacted]								
**Categorized as participated yes/no with no being the reference category**		**Effect size (95%CI)**	**Effect size (95%CI)**	**Effect size (95%CI)**	**Effect size (95%CI)**	**Effect size (95%CI)**	**Effect size (95%CI)**	**Effect size (95%CI)**
Balance exercises	Chinese	**-0.60 (-1.02, -0.17) P=0.006**	0.02 (-1.78, 1.83) P=0.978	-1.64 (-3.66, 0.38) P=0.111	**-1.36 (-2.58, -0.14) P=0.029**	-4.01 (-11.94, 3.93) P=0.323	-0.34 (-1.91, 1.23) P=0.673	-0.37 (-4.10, 3.36) P=0.847
	Malay	-0.63 (-1.94, 0.67) P=0.343	-3.87 (-12.05, 4.32) P=0.355	-3.47 (-8.07, 1.13) P=0.139	**-3.42 (-6.13, -0.71) P=0.014**	-0.30 (-21.19, 20.59) P=0.978	0.25 (-2.82, 3.32) P=0.874	-7.75 (-16.73, 1.22) P=0.090
	Indian	-0.84 (-1.69, 0) P=0.051	-2.25 (-7.07, 2.56) P=0.359	-1.60 (-4.54, 1.33) P=0.284	-0.93 (-2.60, 0.74) P=0.276	3.79 (-10.63, 18.21) P=0.607	**-2.03 (-3.99, -0.07) P=0.042**	-2.49 (-7.99, 3.01) P=0.375
Ball games	Chinese	0.09 (-0.28, 0.46) P=0.638	-0.55 (-2.14, 1.04) P=0.497	-0.09 (-1.78, 1.59) P=0.913	-0.78 (-1.80, 0.23) P=0.131	**-8.87 (-15.62, -2.11) P=0.010**	-0.16 (-1.49, 1.18) P=0.818	1.27 (-1.89, 4.43) P=0.430
	Malay	0.27 (-0.43, 0.97) P=0.451	-3.61 (-8.01, 0.79) P=0.108	0.49 (-1.83, 2.81) P=0.679	**-1.50 (-2.87, -0.13) P=0.032**	2.39 (-8.46, 13.24) P=0.667	0.64 (-0.95, 2.23) P=0.430	-1.45 (-6.12, 3.23) P=0.544
	Indian	0.46 (-0.23, 1.14) P=0.192	-0.28 (-4.17, 3.61) P=0.889	0.67 (-1.67, 3.01) P=0.574	-0.35 (-1.68, 0.98) P=0.610	-4.62 (-16.17, 6.93) P=0.433	-0.36 (-1.93, 1.21) P=0.653	3.89 (-0.52, 8.31) P=0.084
Cycling	Chinese	**-0.80 (-1.23, -0.36) P<0.001**	-1.71 (-3.55, 0.13) P=0.068	-1.84 (-3.81, 0.12) P=0.066	-0.89 (-2.08, 0.29) P=0.140	**-9.40 (-17.22, -1.59) P=0.018**	**1.76 (0.22, 3.31) P=0.026**	-1.56 (-5.21, 2.10) P=0.404
	Malay	-0.70 (-1.44, 0.03) P=0.060	-4.02 (-8.61, 0.58) P=0.087	-0.23 (-2.65, 2.19) P=0.851	0.94 (-0.49, 2.37) P=0.197	-0.67 (-12.03, 10.69) P=0.908	1.06 (-0.60, 2.73) P=0.211	-0.99 (-5.87, 3.90) P=0.693
	Indian	0.37 (-0.61, 1.35)	0.72 (-4.86, 6.30)	0.38 (-2.96, 3.73)	-0.46 (-2.36, 1.45)	7.13 (-9.38, 23.64)	-0.31 (-2.55, 1.93)	1.73 (-4.56, 8.03)

		P=0.458	P=0.800	P=0.822	P=0.637	P=0.397	P=0.787	P=0.589
Dancing	Chinese	0.05<br>(-0.53, 0.64)<br>P=0.857	-0.23<br>(-2.73, 2.27)<br>P=0.855	0.79<br>(-2.01, 3.58)<br>P=0.581	0.25<br>(-1.44, 1.94)<br>P=0.769	-0.74<br>(-11.61, 10.13)<br>P=0.894	**2.49**<br>**(0.34, 4.64)**<br>**P=0.023**	0.43<br>(-4.68, 5.53)<br>P=0.870
	Malay	1.11<br>(-0.43, 2.64)<br>P=0.158	1.87<br>(-7.74, 11.49)<br>P=0.703	0.42<br>(-4.65, 5.49)<br>P=0.871	0.73<br>(-2.26, 3.72)<br>P=0.631	9.35<br>(-14.77, 33.47)<br>P=0.447	-1.31<br>(-4.85, 2.23)<br>P=0.470	-5.65<br>(-16.01, 4.71)<br>P=0.285
	Indian	-0.43<br>(-1.62, 0.75)<br>P=0.475	-2.34<br>(-9.10, 4.43)<br>P=0.499	-2.14<br>(-6.19, 1.91)<br>P=0.301	-2.05<br>(-4.35, 0.25)<br>P=0.081	-1.03<br>(-21.00, 18.95)<br>P=0.920	1.90<br>(-0.81, 4.61)<br>P=0.170	0.39<br>(-7.23, 8.01)<br>P=0.920
Golf	Chinese	0.26<br>(-0.37, 0.90)<br>P=0.414	2.01<br>(-0.69, 4.72)<br>P=0.144	-1.34<br>(-4.26, 1.58)<br>P=0.369	1.43<br>(-0.34, 3.19)<br>P=0.114	5.25<br>(-6.77, 17.27)<br>P=0.392	0.80<br>(-1.58, 3.18)<br>P=0.511	2.29<br>(-3.36, 7.94)<br>P=0.428
	Malay	-0.20<br>(-2.96, 2.55)<br>P=0.885	-1.48<br>(-18.76, 15.80)<br>P=0.867	-4.30<br>(-13.66, 5.06)<br>P=0.368	-1.44<br>(-6.95, 4.07)<br>P=0.609	11.31<br>(-34.47, 57.10)<br>P=0.628	-2.56<br>(-9.29, 4.16)<br>P=0.455	1.66<br>(-18.01, 21.33)<br>P=0.868
	Indian	-0.60<br>(-3.31, 2.10)<br>P=0.661	-0.64<br>(-16.03, 14.75)<br>P=0.935	3.23<br>(-6.22, 12.67)<br>P=0.503	**6.91**<br>**(1.54, 12.28)**<br>**P=0.012**	13.37<br>(-35.12, 61.86)<br>P=0.589	-0.01<br>(-6.60, 6.58)<br>P=0.997	9.15<br>(-9.34, 27.65)<br>P=0.332
Running	Chinese	-0.25<br>(-0.55, 0.05)<br>P=0.097	-1.12<br>(-2.38, 0.13)<br>P=0.080	0.13<br>(-1.22, 1.48)<br>P=0.848	**-1.37**<br>**(-2.19, -0.56)**<br>**P<0.001**	**-6.39**<br>**(-11.76, -1.02)**<br>**P=0.020**	0.43<br>(-0.63, 1.49)<br>P=0.427	-0.83<br>(-3.35, 1.69)<br>P=0.518
	Malay	**-0.63**<br>**(-1.19, -0.08)**<br>**P=0.026**	-1.56<br>(-5.04, 1.92)<br>P=0.379	-1.34<br>(-3.19, 0.51)<br>P=0.155	**-1.18**<br>**(-2.27, -0.09)**<br>**P=0.034**	**-15.59**<br>**(-24.28, -6.90)**<br>**P<0.001**	**1.43**<br>**(0.15, 2.71)**<br>**P=0.028**	**-4.03**<br>**(-7.77, -0.29)**<br>**P=0.035**
	Indian	**-0.67**<br>**(-1.25, -0.08)**<br>**P=0.027**	-2.23<br>(-5.58, 1.12)<br>P=0.192	-0.13<br>(-2.16, 1.90)<br>P=0.900	-0.39<br>(-1.54, 0.76)<br>P=0.509	-9.69<br>(-19.63, 0.26)<br>P=0.056	0.93<br>(-0.42, 2.28)<br>P=0.178	-0.69<br>(-4.48, 3.11)<br>P=0.723
Strength and fitness	Chinese	-0.12<br>(-0.38, 0.13)<br>P=0.345	-1.00<br>(-2.09, 0.10)<br>P=0.074	**1.38**<br>**(0.19, 2.58)**<br>**P=0.023**	-0.02<br>(-0.74, 0.70)<br>P=0.959	-0.31<br>(-5.02, 4.39)<br>P=0.897	**1.85**<br>**(0.92, 2.78)**<br>**P<0.001**	0.70<br>(-1.51, 2.91)<br>P=0.534
	Malay	-0.18<br>(-0.66, 0.30)<br>P=0.461	-0.45<br>(-3.44, 2.55)<br>P=0.770	1.35<br>(-0.27, 2.96)<br>P=0.102	-0.25<br>(-1.21, 0.70)<br>P=0.601	-3.50<br>(-11.03, 4.02)<br>P=0.362	0.81<br>(-0.29, 1.92)<br>P=0.149	1.93<br>(-1.30, 5.17)<br>P=0.242
	Indian	-0.48<br>(-0.98, 0.02)	-1.96<br>(-4.79, 0.87)	1.35<br>(-0.39, 3.08)	0<br>(-0.99, 0.98)	**-11.53**<br>**(-19.99, -3.06)**	**2.12**<br>**(0.97, 3.27)**	-1.75<br>(-4.99, 1.49)

		P=0.058	P=0.176	P=0.128	P=0.992	**P=0.008**	**P<0.001**	P=0.289
Swimming	Chinese	**0.62**<br>**(0.30, 0.94)**<br>**P<0.001**	1.02<br>(-0.35, 2.40)<br>P=0.143	0.68<br>(-0.79, 2.15)<br>P=0.365	**0.95**<br>**(0.06, 1.83)**<br>**P=0.037**	4.51<br>(-1.34, 10.36)<br>P=0.131	-1.01<br>(-2.17, 0.14)<br>P=0.086	1.37<br>(-1.36, 4.11)<br>P=0.325
	Malay	0.53<br>(-0.3, 1.37)<br>P=0.213	**7.33**<br>**(2.09, 12.56)**<br>**P=0.006**	-1.89<br>(-4.65, 0.87)<br>P=0.180	-0.21<br>(-1.84, 1.42)<br>P=0.800	9.31<br>(-3.64, 22.27)<br>P=0.159	-0.44<br>(-2.34, 1.47)<br>P=0.654	-1.06<br>(-6.64, 4.53)<br>P=0.711
	Indian	0.36<br>(-0.43, 1.16)<br>P=0.373	-0.52<br>(5.04, 4.00)<br>P=0.822	-0.03<br>(-2.74, 2.69)<br>P=0.985	0.11<br>(-1.43, 1.66)<br>P=0.885	0.31<br>(-13.13, 13.74)<br>P=0.964	1.25<br>(-0.57, 3.08)<br>P=0.179	3.30<br>(-1.82, 8.43)<br>P=0.207
Walking	Chinese	-0.06<br>(-0.28, 0.16)<br>P=0.601	0.06<br>(-0.88, 0.99)<br>P=0.906	0.01<br>(-1.01, 1.04)<br>P=0.980	0.34<br>(-0.28, 0.96)<br>P=0.285	**4.78**<br>**(0.73, 8.83)**<br>**P=0.021**	0.17<br>(-0.63, 0.97)<br>P=0.680	-0.98<br>(-2.88, 0.92)<br>P=0.311
	Malay	0.02<br>(-0.42, 0.45)<br>P=0.941	0.13<br>(-2.61, 2.87)<br>P=0.926	0.90<br>(-0.58, 2.38)<br>P=0.235	0.59<br>(-0.28, 1.46)<br>P=0.183	-1.80<br>(-8.69, 5.09)<br>P=0.608	0.58<br>(-0.43, 1.60)<br>P=0.258	0.57<br>(-2.39, 3.53)<br>P=0.705
	Indian	-0.07<br>(-0.51, 0.36)<br>P=0.744	-0.90<br>(-3.39, 1.59)<br>P=0.478	0.08<br>(-1.45, 1.60)<br>P=0.923	0.43<br>(-0.44, 1.29)<br>P=0.336	-3.63<br>(-11.11, 3.84)<br>P=0.341	0.81<br>(-0.21, 1.82)<br>P=0.120	0.56<br>(-2.30, 3.41)<br>P=0.702
Other LTPA	Chinese	-0.09<br>(-0.71, 0.54)<br>P=0.783	-0.72<br>(-3.36, 1.92)<br>P=0.593	0.38<br>(-2.47, 3.23)<br>P=0.793	-0.83<br>(-2.55, 0.89)<br>P=0.345	-0.66<br>(-11.99, 10.67)<br>P=0.909	-0.02<br>(-2.26, 2.22)<br>P=0.987	0.11<br>(-5.19, 5.41)<br>P=0.967
	Malay	0.38<br>(-0.49, 1.26)<br>P=0.393	2.79<br>(-2.71, 8.28)<br>P=0.320	-1.06<br>(-3.95, 1.83)<br>P=0.473	-1.33<br>(-3.03, 0.38)<br>P=0.127	-6.10<br>(-19.66, 7.47)<br>P=0.378	0.77<br>(-1.22, 2.76)<br>P=0.449	2.42<br>(-3.43, 8.26)<br>P=0.417
	Indian	0.64<br>(-0.57, 1.84)<br>P=0.302	0.80<br>(-6.07, 7.67)<br>P=0.820	1.50<br>(-2.63, 5.63)<br>P=0.477	-0.15<br>(-2.50, 2.20)<br>P=0.899	-3.19<br>(-23.41, 17.04)<br>P=0.758	1.03<br>(-1.72, 3.78)<br>P=0.461	-1.14<br>(-8.85, 6.58)<br>P=0.773

* Model adjusted for age, gender, ethnicity, educational status, smoking status, alcohol consumption and other exercise subtypes. CI, confidence interval; BMI, body mass index; HDL, high-density lipoprotein; LDL, low-density lipoprotein; LTPA, leisure time physical activity. Bold values indicate statistical significance.
